# Mesh closure of a protruding caesarean scar pregnancy in the 11th week of pregnancy

**DOI:** 10.1186/s12884-026-09544-w

**Published:** 2026-07-03

**Authors:** Mehmet Vural, Maximillian Rauh, Antigoni Hadjiiona, Tilman Born, Angela Köninger

**Affiliations:** Department of Obstetrics and Gynecology, University of Regensburg, Clinic St. Hedwig, Steinmetzstrasse 1-3, Regensburg, 93049 Germany

**Keywords:** Scar pregnancy, Conservative operation, Mesh closure

## Abstract

We report the case of a 30-year-old woman in the first trimester with a cesarean scar pregnancy (CSP) following a previous caesarean section, who wished to preserve the pregnancy. External advice had previously recommended termination, but an individualized, experimental approach was agreed upon. At 11th weeks’ gestation, a mesh was placed over the scar dehiscence and secured in position. The uterus was strongly retroflexed, and the placenta prolapsed through the scar. Direct closure of the cranial and caudal myometrium surrounding the protruding placenta was not feasible; the defect measured approximately 4 cm, and the prolapsed placenta was the size of a mandarin. The patient was closely monitored until 32 weeks’ gestation, when she presented with lower abdominal pain and mild vaginal bleeding. A hysterectomy was performed at the patient’s request, although a focal resection had been offered. This case demonstrates that live birth can be achieved in extreme CSP and suggest that mesh coverage of large cesarean scar defects may represent a novel, pregnancy-preserving therapeutic option.

## Introduction

Caesarean scar pregnancy (CSP) is an early pregnancy complication in which the placenta implants into the previous cesarean scar. In the non-pregnant state, this may be identified as a niche within the uterine scar. When diagnosed in early gestation, CSP can be managed; however, in later weeks, it frequently progresses to placenta accreta spectrum disorder (PASD). These three entities – niches, CSP and PASD- share a common pathology involving disruption of normal myometrial anatomy. Each condition carries distinct clinical manifestations and complications: niche formation is associated with infertility, abnormal bleeding, chronic pelvic pain [1]; CSP carries a high risk of miscarriage and hemorrhage; and PASD is linked to preterm birth and maternal morbidity and mortality. Histological studies have shown that cesarean scar pregnancy (CSP) and placenta accreta spectrum disorder (PASD) arising from scar implantation and uterine dehiscence share similar histopathological features and may be difficult to distinguish histologically [2]. However, PASD can also occur independently of CSP.CSPs may be preventable even before conception. The primary risk factor is a previous cesarean section (CS), with the risk of abnormal scarring increasing with multiple CS [3]. PASD has also been reported following myomectomy or other uterine procedures [4]. If a uterine niche is identified preconceptually, surgical repair of the scar may be considered to reduce the risk of CSP [5]. In contrast, surgical repair during pregnancy is uncommon and considered highly experimental. In many cases, gestation can be managed expectantly, allowing the pregnancy to continue despite the anticipated development of placenta percreta at term [6]; however, termination is widely recommended to prevent uterine rupture [7]. In cases with extensive placental protrusion, as illustrated in the present case, a pregnancy-preserving approach poses a significant clinical challenge.

## Case report

**Fig. 1:**
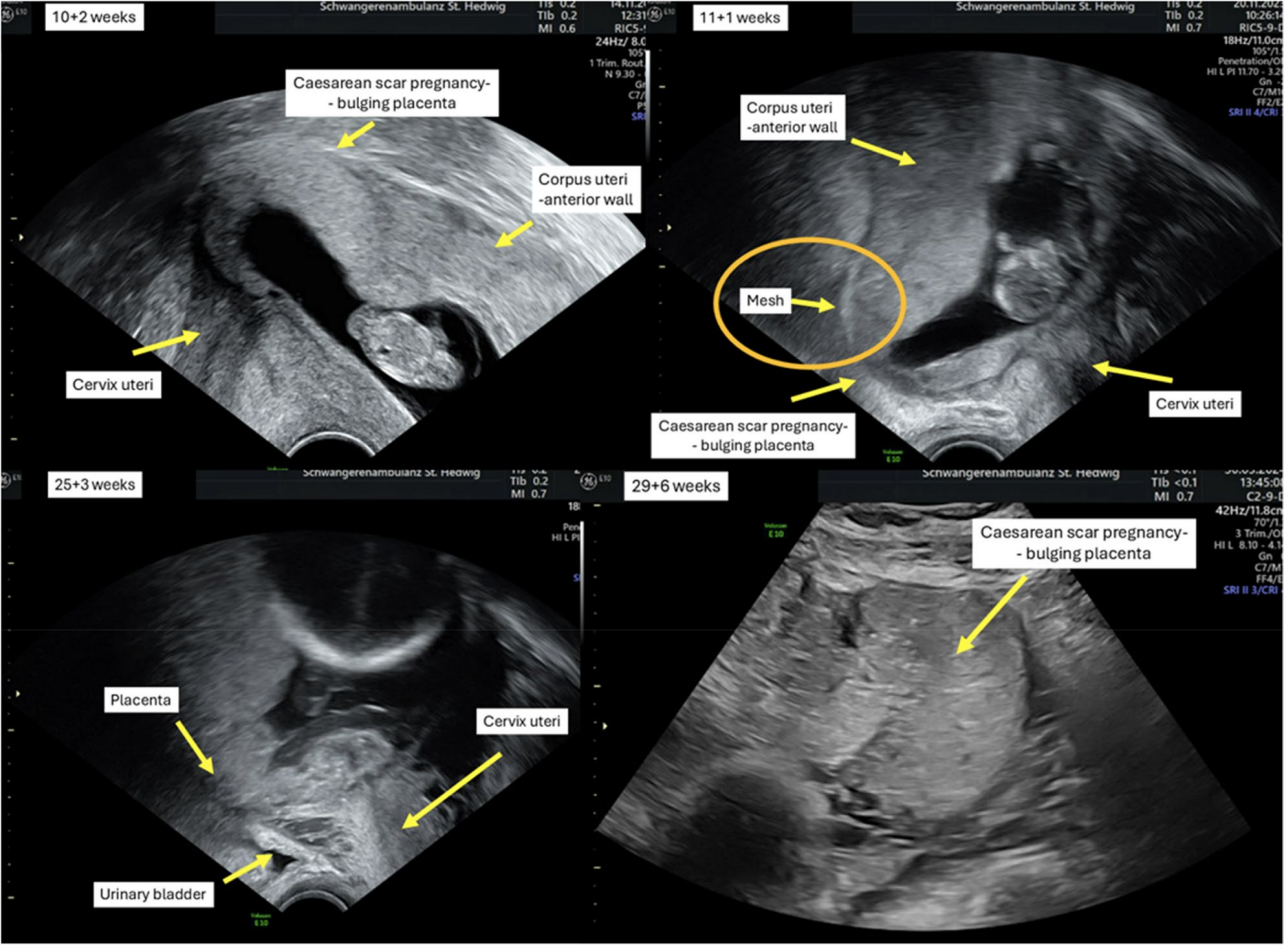
Transvaginal ultrasound images demonstrating uterine wall defects and implanted mesh

**Fig. 2:**
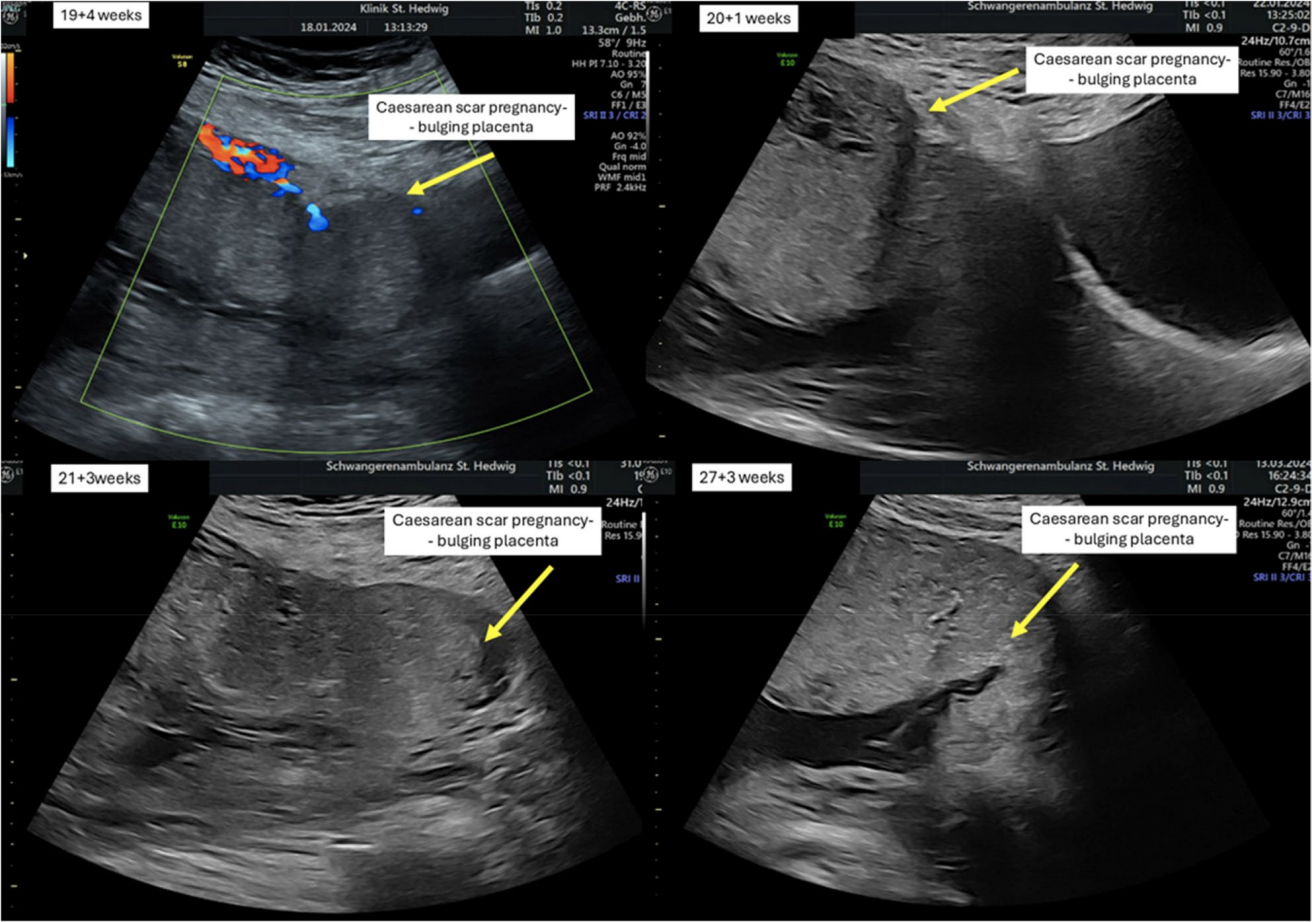
Abdominal ultrasound images demonstrating the bulging placenta at different gestational ages

A 30-year-old woman in the first trimester presented with a CSP complicated by a high-grade placental protrusion extending through the uterus dehiscence towards outside. She conceived via in vitro fertilization (IVF) and expressed a strong desire to preserve the pregnancy, having previously declined termination at another clinic. As she first presented during an ongoing pregnancy, there was no opportunity for preconception correction of the uterine defect. At 11 weeks’ gestation, the ectopic scar pregnancy demonstrated an exogenous placental bulge, placing her at high risk for uterus rupture without preventive intervention. No hemorrhage was present at the time of presentation. Imaging demonstrated an anterior uterine wall defect measuring approximately 4 cm in the sagittal plane, with a persistently bulging placenta observed on all ultrasound examinations (Figs. 1 and 2). The crown-rump-length of the fetus was 35.2 mm and distal to the defect was unaffected and of normal length.

After extensive counseling, an individualized and experimental approach was agreed upon. The plan involved open closure following diagnostic laparoscopy, with the possible use of a mesh to support myometrial proliferation and encourage the placenta to reposition into the uterine cavity. The patient was informed that, given the advanced gestational age and the complexity of her presentation, this procedure was highly experimental and should be considered only as an alternative to pregnancy termination.

**Fig. 3:**
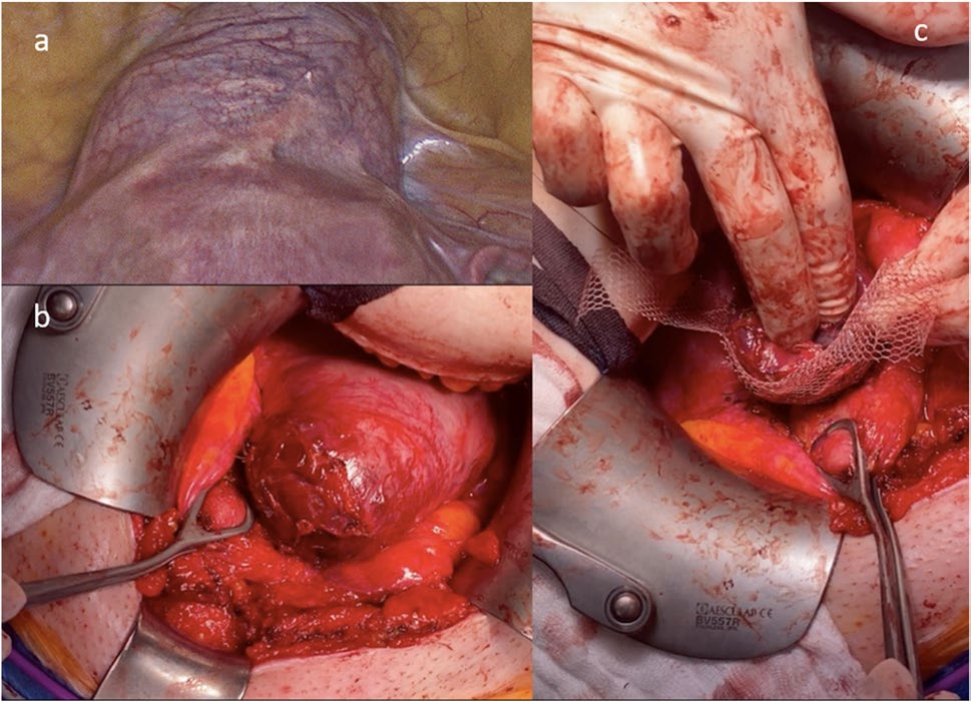
Intraoperative images showing uterine surgery and mesh implantation. **a** Laparoscopic view of the anterior uterine wall and the bladder fold. **b** Intraoperative view showing the uterine wall defect after surgical preparation. **c** Intraoperative placement of a mesh over the uterine defect. The surgeon positions the mesh, which will reinforce the uterine wall

In addition, the findings were so extensive that, even at this stage, suction evacuation or treatment with methotrexate was considered infeasible in term of achieving surgical repair. Instead, excision of the percreta area—analogous to the approach used in advanced stages of pregnancy—was considered the only viable option. The patient was therefore informed that the same surgical procedure would be required regardless of gestational age. Diagnostic laparoscopy was performed to determine the optimal surgical approach. This step was also essential to assess potential placental attachment to the anterior abdominal wall and to guide the incision for the planned open surgery. Intraoperatively, the placenta was observed prolapsing through the dehiscent isthmocervical region. No adhesions to the anterior abdominal wall were identified that would have precluded a transverse laparotomy. These findings supported the feasibility of a transverse abdominal approach, enabling careful manipulation and repositioning of the protruding placental tissue into the uterine cavity while minimizing the risk of inadvertent placental disruption and hemorrhage (Fig. 3). 

### Transverse laparotomy and scar closure with a mesh

**Fig. 4:**
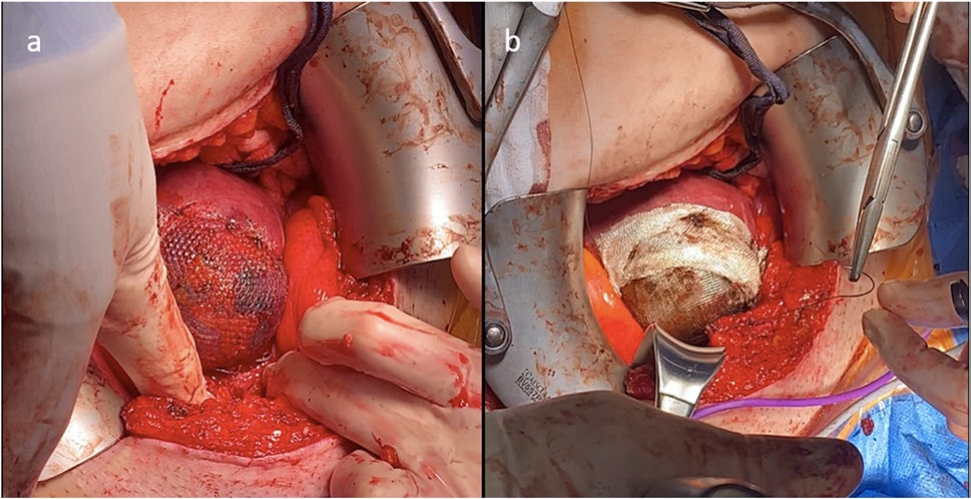
Intra-operative images showing the final steps of mesh placement for uterine wall defect repair. **a** The uterine wall is reinforced with a pre-shaped mesh (InGYNious anterior large AMI®) which has been secured over the uterine defect. The surgeon ensures proper placement and tension of the mesh to fully cover the defect and provide structural support to the compromised uterine tissue. **b** Final adjustments of the mesh, including circumferential fixation of a Tabotamp® patch over the implanted mesh, secured with absorbable sutures to cover the defect as completely as possible

Unfortunately, the defect was too extensive to allow the suturing of the caudal and cranial borders of the myometrium above and below the defect without risking miscarriage, as the placenta had prolapsed too far. Therefore, a mesh (InGYNious^®^ anterior large, AMI^®^) was placed over the placenta and secured to the cervix and the cranial margin of the intact myometrium. Prior to mesh fixation, the placenta was gently repositioned into the uterine cavity manually as far as possible. This maneuver was surprisingly effective and resulted in the uterus assuming an anteflexed position. The mesh was then attached circumferentially at its borders to the cervix and adjacent intact myometrium using many interrupted Ethibond^®^ 3 − 0 sutures, which were individually tied to secure the mesh in place. At the end of the procedure, fetal cardiac activity was present. A tabotamp^®^ patch was then placed between the posterior vaginal wall and the cervix or prolapsing placenta (Figs. 3 and 4).

### CS in the 32nd week of pregnancy

**Fig. 5:**
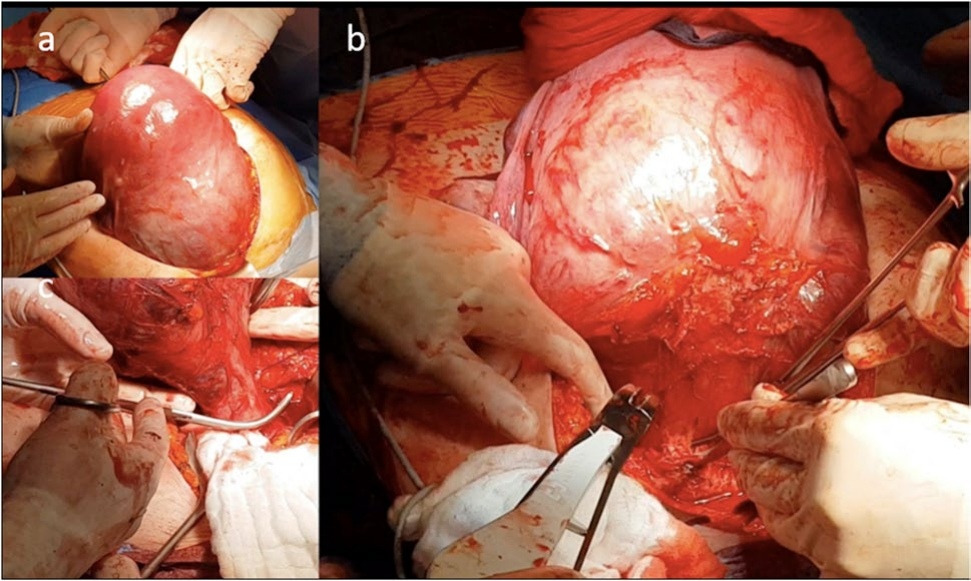
Intraoperative images illustrating cesarean hysterectomy. **a** Exteriorized uterus at the beginning of the cesarean hysterectomy; the fetus remains in situ, and the uterus is fully mobilized for the procedure. **b** Uterine preparation following delivery of the fetus via a longitudinal fundal incision. **c** Final step showing the uterus detached from surrounding structures, ready for complete surgical removal (hysterectomy)

**Fig. 6:**
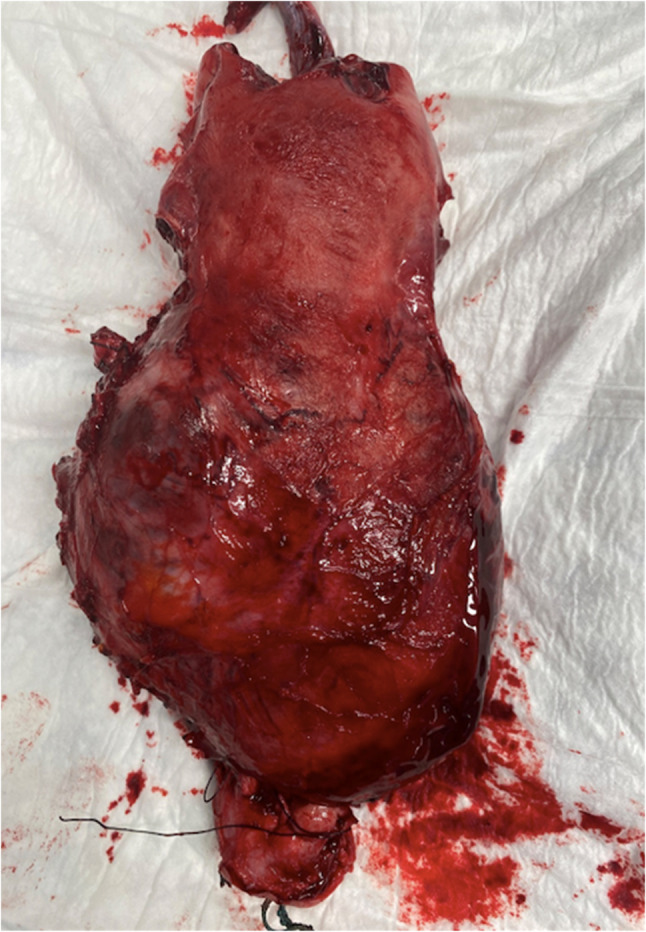
Excised uterus following cesarean hysterectomy. The uterus has been completely removed. The implanted mesh is visible within the deeper layers of the anterior uterine wall

Throughout the pregnancy, recurrent placental protrusion was noted without acute complication. At 21 weeks’ gestation, the patient was admitted with abdominal pain and treated with calcium channel blockers to achieve uterine relaxation in view of persistent risk of uterine rupture. Owing to vaginal bleeding and increasing lower abdominal pain, a caesarean delivery was indicated. At the patient’s request, a postpartum hysterectomy was performed. Intraoperatively, there was no evidence of uterine rupture or thinning of myometrium, although the previously placed mesh was noted deeply embedded within the anterior uterine wall (Figs. 5 and 6). A focal resection of the percreta area and primary scar closure-preserving the future fertility-was offered but declined by the patient. Access was achieved via a longitudinal laparotomy, and the infant was delivered through a fundal uterotomy. A supracervical hysterectomy was completed without the need for blood transfusion. The procedure was uneventful, and the patient’s hemoglobin at the end of surgery was 9.9 mg/dL.

Delivery was performed at 31 + 1 weeks of gestation, resulting in a healthy female newborn. The infant had a birth weight of 1990 g, a length of 40 cm, an Apgar score of 2/8/9, and an arterial umbilical cord pH of 7.34. She was discharged after an uneventful neonatal course on day 32 after birth. Estimated blood loss was 900 ml.

## Discussion

CSP is a complication of the early pregnancy for which an optimal therapeutic strategy has not yet been defined. In this case report, we present a challenging approach and demonstrate the extent to which this condition may be managed conservatively in selected cases.

Two main types of CSP have been described: endogenous, in which the gestational sac grows toward the uterine cavity, and exogenous, in which the pregnancy grows outward toward the serosa and abdominal cavity [8]. In exogenous CSP, cesarean hysterectomy is frequently performed [9]. Alternatively, focal resection for PASD may be considered after dissection of the bladder from the anterior uterine wall [10]. However, our patient preferred a hysterectomy.

Among the various diagnostic methods, transvaginal ultrasound examination is the most commonly used and reliable method for evaluating cesarean scar pregnancy [11]. In our case, it also allowed clear visualization of the fixed mesh (Fig. 1).In our case, despite embryo transfer to the fundus, implantation occurred within the niche. This case illustrates the potential risk associated with cesarean scar niches in fertility patients and may support consideration of preconception evaluation and, in selected cases, surgical correction. In our experience, the conservative management of CSP remains a viable option, although it is not recommended by the Society for Maternal-Fetal Medicine (SMFM) [12]. We have previously reported a case in which a protruding placenta and cesarean scar dehiscence were successfully managed via robotic surgery [13]. Based on these findings, we believe that conservative management of CSP with an ongoing pregnancy should be considered and may become increasingly feasible with growing clinical experience. Not all woman have the opportunity for timely subsequent conception, and in some cases-such as ours-pregnancy may be achieved through assisted reproductive technologies. Regardless of etiology, medical history, or whether placental growth is endogenous or exogenous, we advocate offering the conservative approach with preservation of the pregnancy, provided that patients receive comprehensive counseling regarding the associated risks.

To date, treatment options for CSP include local or systemic administration of methotrexate, hysteroscopic or laparoscopic resection, vaginal approaches, dilation and curettage, aspiration, uterine artery embolization, high-intensity focused ultrasound (HIFU), or a combination of these modalities [14]. Notably, even within this series, expectant management was associated with a complication rate exceeding 50%, including severe hemorrhage and the need for hysterectomy.

Our method was experimental but appeared to be effective in this case. Therefore, mesh placement may represent a potential alternative for patients with a markedly prolapsing placenta who wish to preserve both the pregnancy and the uterus. 

## Data Availability

The authors confirm that the data supporting the findings of this study are available within the article.
